# Characteristics of resuscitation, and association between use of dynamic tests of fluid responsiveness and outcomes in septic patients: results of a multicenter prospective cohort study in Argentina

**DOI:** 10.1186/s13613-020-00659-7

**Published:** 2020-04-15

**Authors:** Arnaldo Dubin, Cecilia Loudet, Vanina S. Kanoore Edul, Javier Osatnik, Fernando Ríos, Daniela Vásquez, Mario Pozo, Bernardo Lattanzio, Fernando Pálizas, Francisco Klein, Damián Piezny, Paolo N. Rubatto Birri, Graciela Tuhay, Analía García, Analía Santamaría, Graciela Zakalik, Cecilia González, Elisa Estenssoro, the investigators of the SATISEPSIS group, Carolina Enrico, Mariel Romitelli, Mariel Ayelén García, José Celia, Leandro Machuca, Fernando Pálizas, Mario Pozo, Bernardo Lattanzio, Emanuel Valgolio, Mario Kenar, Carlos Sosa, Sergio Sarquis, Graciela Tuhay, Francisco Klein, Ariel Sosa, Daniel Ivulich, Luciana Bianchi, Enrique Ruben Correger, Carla Groer, Ma. Victoria Arrosagaray, Graciela Cueto, Carlos Cozzani, Gustavo Badariotti, Bernardo de Diego, Daniela Vasquez, Gustavo Plotnikov, Analía Santa María, Mariana Bertes, Alejandro Gomez, María Soledad Santagiuliana, Margarita Tavela, Pierina Bachetti, Célica Irrazabal, Alejandro Risso Vazquez, Paolo Nahuel Rubatto Birri, Gabriel Olarte, Veronica Marcela Cannatelli, Anatilde Díaz, Analía García, Estefanía Minoldo, Cayetano Galletti, Esteban Payer, Marcelo Avilez, Silvio Ernesto Lazzeri, Luis A. Huespe, Lorena de los Ángeles Parra, Fernando Kurban, Carlos Anibal Pellegrini, Adrian Alfredo Martin, Graciela Zakalik, Magalí Sanchez, Natalia Barreto, Alfredo Elías Carreras, Johana Bastias, Julián Ivacachi, María Luz Campassi, Fabio Germán Repetto, María Gabriela Saenz, Cecilia Marchena, María Rosa Marino, Gerardo Ezcurra, Sebastián Caravaggio, María de los Ángeles García, Ana María Mazzola, Analía Piernatei, Estela Molinas, Mauro Iadanza, Mario Alejandro Traba, Leda Fiorella Bacci, Adriana Fernandez, Damián Piezny, Constanza Arias, Gustavo Chaparro, Graciela Celeste Lopez, Agustín Fernández, Catalina Reyes Najera, Adriana Baldiviezo, Alejandra Flores, Alejandro Risso Vazquez, Irma Moyano, Mónica Quinteros, Laura Budrovich, Lilen Corzo, Sebastián Andrés Amieva, Melisa Ré, Nicolás Rocchetti, Juan Carlos Pendino, Lisandro Bettini, Lionel Talamonti, Gustavo Izaguirre, German Schmukler, Ignacio Sabbag, Tomas F. Diez, Laura Bergallo, Cecilia González, Carlos Lovesio, Daniel Duarte, Romina Nicastro, Fernando Bertoletti, Esteban Milioto

**Affiliations:** 1 Servicio de Terapia Intensiva, Sanatorio Otamendi, Azcuénaga 870, C1115 AAB Buenos Aires, Argentina; 2 Hospital Interzonal de Agudos San Martin de La Plata, La Plata, Buenos Aires Argentina; 3 Hospital Juan A Fernández, Buenos Aires, Argentina; 4 Hospital Alemán, Buenos Aires, Argentina; 5 Hospital Alejandro Posadas, El Palomar, Buenos Aires, Argentina; 6 Sanatorio Anchorena, Buenos Aires, Argentina; 7 Clínica Bazterrica, Buenos Aires, Argentina; 8 Clínica Santa Isabel, Buenos Aires, Argentina; 9 Hospital Universitario Fundación Favaloro, Buenos Aires, Argentina; 10 Hospital Misericordia, Córdoba, Argentina; 11 Sanatorio de la Trinidad Mitre, Buenos Aires, Argentina; 12 Hospital Lagomaggiore, Mendoza, Argentina; 13 Sanatorio Parque, Rosario, Santa Fe Argentina

**Keywords:** Sepsis, Hypoperfusion, Vasopressors, Fluid responsiveness, Dynamic tests

## Abstract

**Background:**

Resuscitation of septic patients regarding goals, monitoring aspects and therapy is highly variable. Our aim was to characterize cardiovascular and fluid management of sepsis in Argentina, a low and middle-income country (LMIC). Furthermore, we sought to test whether the utilization of dynamic tests of fluid responsiveness, as a guide for fluid therapy after initial resuscitation in patients with persistent or recurrent hypoperfusion, was associated with decreased mortality.

**Methods:**

Secondary analysis of a national, multicenter prospective cohort study (*n* = 787) fulfilling Sepsis-3 definitions. Epidemiological characteristics, hemodynamic management data, type of fluids and vasopressors administered, physiological variables denoting hypoperfusion, use of tests of fluid responsiveness, and outcomes, were registered. Independent predictors of mortality were identified with logistic regression analysis.

**Results:**

Initially, 584 of 787 patients (74%) had mean arterial pressure (MAP) < 65 mm Hg and/or signs of hypoperfusion and received 30 mL/kg of fluids, mostly normal saline (53%) and Ringer lactate (35%). Vasopressors and/or inotropes were administered in 514 (65%) patients, mainly norepinephrine (100%) and dobutamine (9%); in 22%, vasopressors were administered before ending the fluid load. After this, 413 patients (53%) presented persisting or recurrent hypotension and/or hypoperfusion, which prompted administration of additional fluid, based on: lactate levels (66%), urine output (62%), heart rate (54%), central venous O_2_ saturation (39%), central venous–arterial PCO_2_ difference (38%), MAP (31%), dynamic tests of fluid responsiveness (30%), capillary-refill time (28%), mottling (26%), central venous pressure (24%), cardiac index (13%) and/or pulmonary wedge pressure (3%). Independent predictors of mortality were SOFA and Charlson scores, lactate, requirement of mechanical ventilation, and utilization of dynamic tests of fluid responsiveness.

**Conclusions:**

In this prospective observational study assessing the characteristics of resuscitation of septic patients in Argentina, a LMIC, the prevalent use of initial fluid bolus with normal saline and Ringer lactate and the use of norepinephrine as the most frequent vasopressor, reflect current worldwide practices. After initial resuscitation with 30 mL/kg of fluids and vasopressors, 413 patients developed persistent or recurrent hypoperfusion, which required further volume expansion. In this setting, the assessment of fluid responsiveness with dynamic tests to guide fluid resuscitation was independently associated with decreased mortality.

## Background

Fluids and vasopressor therapy is the cornerstone of the hemodynamic resuscitation of septic shock. Nevertheless, the proper management of these therapeutic tools remains controversial, including the volume and the type of fluid infused, the choice of the vasoconstrictor and the timing of its beginning, as well as the appropriate monitoring for deciding and tracking each intervention. Despite these conflictive standpoints, the resuscitation bundles of the Surviving Sepsis Campaign (SSC) are commonly accepted [1]. Thus, the presence of hemodynamic instability or tissue hypoperfusion is a usual trigger for the intravenous administration of 30 mL/kg of crystalloid solutions. SSC recommendations also include the infusion of norepinephrine to reach a mean arterial pressure (MAP) of at least 65 mm Hg. If manifestations of hypoperfusion persist after the fluid load, further intravascular volume expansion and eventually vasopressors and inotropes should be considered to optimize cardiovascular performance [1].

Despite the fact that the compliance with the SSC has been associated with improved outcomes [2, 3], aggressive fluid administration and its consequence of volume overload can increase the mortality of septic patients [4–7]. Given that both insufficient and excessive administration of solutions might be harmful, fluid management in sepsis should be carefully optimized. For this purpose, the use of dynamic predictors of fluid responsiveness might be especially beneficial [8–10]. These tests anticipate that a fluid challenge will result in an increase of cardiac output > 15%, without the risk of volume overload. Although a recent study showed its feasibility during the early resuscitation of septic shock [11], the approach is still scarcely used to assess the appropriateness of a fluid challenge [12].

SATISEPSIS was an observational study organized by the Argentine Society of Critical Care (Sociedad Argentina de Terapia Intensiva, SATI) to evaluate the performance of the new Sepsis-3 definitions [13], and to characterize the epidemiology and outcome of sepsis and septic shock in Argentina, a country belonging to the group of low and middle-income countries (LMICs) [14, 15]. The aim of the present study was to assess the characteristics of the cardiovascular management, particularly regarding early resuscitation and use of intravenous fluids. A particular hypothesis we sought to test was if the utilization of dynamic predictors of fluid responsiveness, as a guide for fluid therapy after the initial fluid resuscitation, is associated with a reduced mortality.

## Methods

### Design

Briefly, SATISEPSIS was a national, multicenter, prospective cohort study organized by the SATI and sponsored by the Argentine National Ministry of Health, beginning on July 2, 2016 and lasting for 3 months [14, 15]. The study was approved by each hospital’s Institutional Review Board, and informed consent was signed by patients or their relatives.

### Patients

Patients included were ≥ 18 years, admitted to the 49 participating ICUs with a suspected infection that triggered blood cultures and/or other body fluid sampling, and administration of antibiotics within 24 h. Patients with infections developed during ICU stay were also considered. Patients were characterized as having sepsis or septic shock according to Sepsis-3 definitions [13]. Epidemiological data, Charlson, APACHE II and SOFA scores, use and length of mechanical ventilation, complications, and ICU and hospital length of stay were also recorded.

The main outcome variable was hospital mortality.

*Assessment of initial resuscitation during the first 24* *h* On protocol admission, initial evaluation consisted in the identification of arterial hypotension (MAP < 65 mm Hg) and/or the evidence of hypoperfusion, defined as: blood lactate levels > 2.0 mmol/L, oliguria, capillary-refill time (CRT) > 3 s, and presence of mottling. We recorded if the initial fluid load of 30 mL/kg recommended by the SSC was administered [1]. The type of fluid used was recorded, as 0.9% NaCl (normal saline, NS), Ringer lactate, Ringer acetate, and colloids (albumin, gelatin and starch solutions).

After this, researchers were asked if hypotension was corrected (MAP ≥ 65 mm Hg), if other variables showed an insufficient response (Table 1), and if vasopressors and/or inotropes had to be added. The type of drug used was registered. Over the first 24 h, researchers were asked to assess the presence of persistent or recurrent signs of hypoperfusion, and whether additional volume expansion after the initial resuscitation with 30 mL/kg of fluids was considered. If so, we asked them to identify the variables that supported the additional fluid administration in this subgroup, which were: MAP, heart rate, central venous pressure, lactate, central venous oxygen saturation, central venous–arterial PCO_2_ difference, urine output, CRT, skin mottling, cardiac output, pulmonary wedge pressure, central venous pressure, and tests for dynamic assessment of fluid responsiveness. These last included the respiratory variation of arterial pulse pressure, systolic volume, and pulse oximetry plethysmographic waveforms; the respiratory variation of inferior vena cava diameter; and the passive leg-raising maneuver. Description of these tests with their cutoff points are shown in Additional file 1.

**Table 1:** Variables used to assess the response to the initial fluid bolus of 30 mL/kg

Variable	Cutoff value for considering an insufficient response
Mean arterial pressure	< 65 mm Hg
Heart rate	> 120 beats/min
Central venous pressure	> 6 mm Hg
Lactate clearance	< 20%/2-h
Central venous O_2_ saturation	< 70%
Central venous–arterial PCO_2_ difference	> 6 mm Hg
Urine output	< 0.5 mL/kg/h
Capillary-refill time	> 3.0 s
Skin mottling	Present

### Statistical analysis

Data are presented as proportions, mean and standard deviation, or median and interquartile range [25–75%]. Differences between survivors and nonsurvivors were analyzed in the entire group, and also in the patients of the subgroup with hypoperfusion requiring additional fluids after initial resuscitation. Chi-square or Fisher tests, *t* or Wilcoxon rank-sum tests, were utilized, as appropriate. No assumptions were made for missing data.

In the subgroup of patients with MAP < 65 mm Hg and/or hypoperfusion that required additional fluids after initial resuscitation, we sought to identify independent predictors of hospital mortality. Variables differing between survivors and nonsurvivors with a *P* value < 0.20 were entered into a multivariable regression model. Model calibration was assessed using the Hosmer–Lemeshow test. A receiver operating characteristic (ROC) curve was built to assess model discrimination. The possibility of an effect of the different centers on mortality was tested in a mixed-effect model, with the variable hospital as the random term.

## Results

**Fig. 1:**
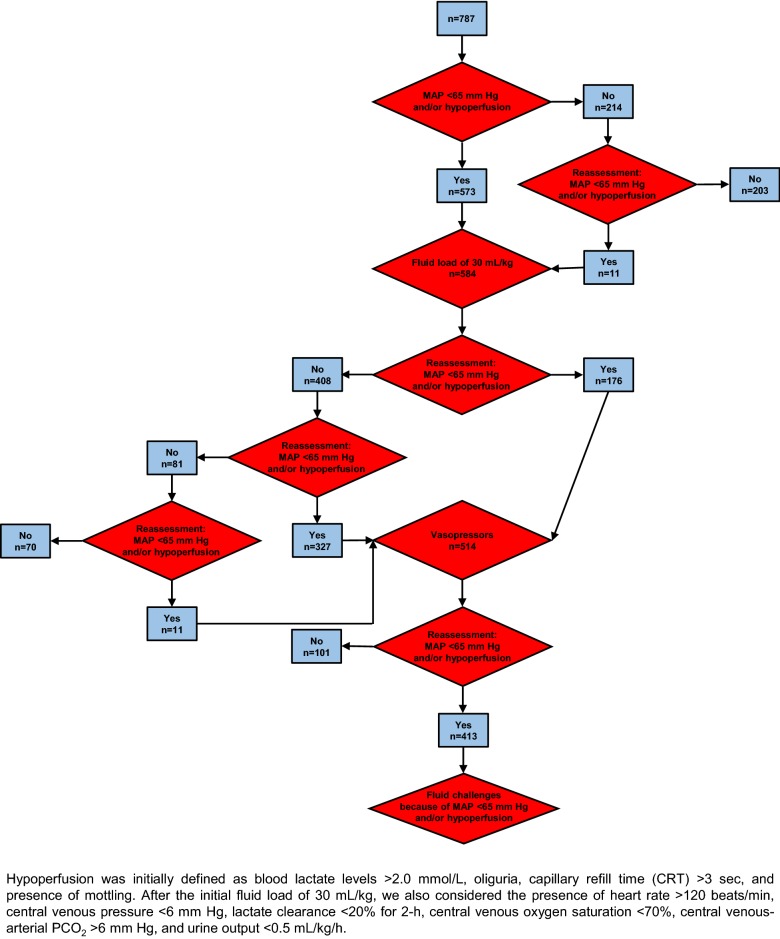
Flowchart of the study

Of the 809 patients included in the study, 787 (97%) had hemodynamic management data. The flowchart of patients is shown in Fig. 1. Epidemiological and hemodynamic variables and comparisons between survivors and nonsurvivors are shown in Additional file 1: Table S1. Hospital mortality was 37%.

At inclusion in the study, 573 of 787 of patients (73%) exhibited a MAP < 65 mm Hg and/or signs of hypoperfusion; all received 30 mL/kg of fluids as initial resuscitation. Additionally, 11 patients not hypotensive at inclusion, when reevaluated had a decrease of MAP to < 65 mm Hg, thus also received the fluid bolus.

**Fig. 2:**
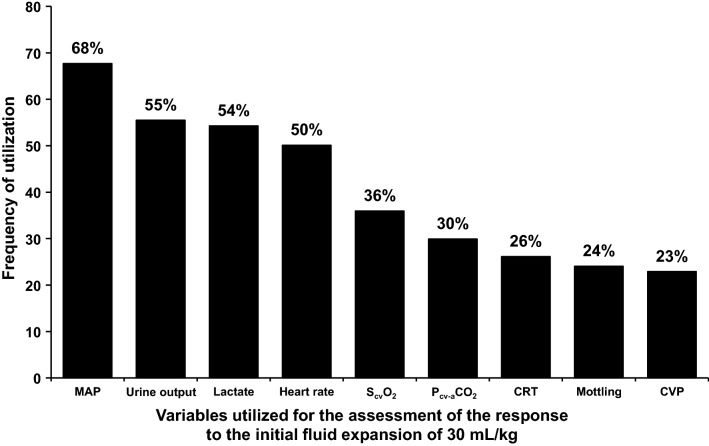
Frequency of the variables utilized as criteria of hypoperfusion for the assessment of the response to the initial fluid bolus of 30 mL/kg. MAP, mean arterial pressure; S_cv_O_2_, central venous O_2_ saturation; P_cv-a_CO_2_, central venous–arterial PCO_2_ difference; CRT, capillary-refill time; CVP, central venous pressure

After this initial fluid load, 176/584 of the patients (30%) remained hypotensive and received vasopressors. The rest of the patients (408/584, 70%) reached a MAP > 65 mm Hg. However, when subsequently reassessed, 349 of the 584 (60%) were hypotensive or had signs of hypoperfusion, and 338/584 (58%) of them required vasopressors. The sum of these two subgroups requiring vasopressors (*n* = 514) was reevaluated for MAP < 65 mmHg and/or signs of persistent or recurrent hypoperfusion, which was diagnosed utilizing clinical, hemodynamic, and biochemical measurements (Fig. 2). MAP < 65 mm Hg and/or persistent or recurrent hypoperfusion developed in 413 of these patients, prompting the infusion of additional fluid boluses. This subgroup had higher mortality compared to patients that did not require additional fluid loads (48% vs. 37%, *P* < 0.0001) and is further characterized in Table 2.

**Table 2:** Characteristics of the patients with persistent or recurrent hypoperfusion after the initial fluid load and vasopressor administration, and who required additional fluid administration

	All (*n* = 413)	Survivors (*n* = 216)	Nonsurvivors (*n* = 197)	*P*-value
Age (years)	60 ± 18	58 ± 19	62 ± 17	0.007
Gender, female	196/413 (47)	111/216 (51)	85/197 (43)	0.09
Charlson score	2 [0–4]	1 [0–3]	3 [1–4]	< 0.000
Previous duration of symptoms	48 [16–72]	24[12–72]	48 [24–96]	< 0.000
Admission to a public hospital	211/413 (51)	85/216 (39)	126/197 (64)	< 0.000
APACHE II score	21 ± 9	18 ± 8	24 ± 9	< 0.000
Systemic inflammatory response syndrome	385/409 (94)	206/215 (96)	179/194 (92)	0.13
Shock on admission	216/409 (51)	90/216 (42)	126/197 (64)	< 0.000
SOFA score	8 ± 4	7 ± 4	10 ± 4	< 0.000
Arterial lactate (mmol/L)	2.5 [1.6–4.0]	2.1 [1.4–3.5]	2.8 [1.9–5.1]	< 0.000
Utilization of mechanical ventilation	280/386 (73)	109/202 (54)	171/184 (93)	< 0.000
Time to the first antibiotic dose (h)	2 [1–5]	2 [1–4]	3 [1–6]	0.004
Complications				
Acute respiratory distress syndrome	131/385 (34)	52/200 (26)	79/185 (43)	0.89
Gastrointestinal bleeding	10/385 (3)	5/201 (2)	5/184 (3)	0.94
Length of mechanical ventilation (days)	4 [0–10]	2 [0–8]	5 [2–11]	< 0.0001
ICU length of stay (days)	8 [3–18]	10 [4–20]	6 [2–12]	< 0.0002
Hospital length of stay (days)	17 [8–33]	23 [13–40]	10 [3–23]	< 0.0001

Data are shown as number (percentage), median [IQR] or mean ± SD

The need of subsequent infusion of fluid boluses was evaluated with different variables and tests of fluid responsiveness (Table 3 and Additional file 1: Figure S1).

**Table 3:** Variables used to support the additional fluid challenges in patients with persistent or recurrent hypoperfusion after the initial fluid load of 30 mL/kg

	All (*n* = 413)	Survivors (*n* = 216)	Nonsurvivors (*n* = 197)	*P*
Mean arterial pressure	127/413 (31)	68/216 (31)	59/197 (30)	0.736
Heart rate	222/413 (54)	120/216 (56)	102/197 (52)	0.442
Central venous pressure	100/413 (24)	47/216 (22)	53/197 (27)	0.223
Lactate	274/413 (66)	142/216 (66)	132/197 (67)	0.786
Central venous O_2_ saturation	164/413 (39)	90/216 (42)	74/197 (38)	0.395
Central venous–arterial PCO_2_ difference	156/413 (38)	77/216 (36)	79/197 (40)	0.351
Urine output	257/413 (62)	137/216 (63)	120/197 (61)	0.599
Capillary-refill time	117/413 (28)	63/216 (29)	54/197 (27)	0.693
Presence of mottling	108/413 (26)	52/216 (24)	56/197 (28)	0.315
Cardiac output measurement	53/413 (13)	24/216 (11)	29/197 (15)	0.273
Pulmonary wedge pressure	11/413 (3)	3/216 (1)	8/197 (4)	0.08
Dynamic tests of fluid responsiveness	122/413 (30)	73/216 (34)	49/197 (25)	0.047
Pulse pressure variation	85/413 (21)	50/216 (23)	35/197 (18)	0.17
Systolic volume variation	33/413 (8)	19/216 (9)	14/197 (8)	0.527
Pulse oximetry waveform variation	25/413 (6)	18/216 (8)	7/197 (14)	0.04
Inferior vena cava diameter variation	52/413 (13)	31/216 (14)	2321/197 (11)	0.259
Passive leg raising	38/413 (9)	19/216 (9)	19/197 (10)	0.766
Other	31/413 (8)	20/216 (9)	11/197 (6)	0.157

Data are shown as number (percentage)

Different vasopressor and/or inotropic drugs were administered in 514 (65%) patients, especially norepinephrine and dobutamine (Additional file 1: Figure S2). In 113 patients (22%), norepinephrine was started before finishing the initial fluid load. Normal saline was utilized in 53% of patients; Ringer lactate in 36%. Colloids were used in 7% of patients (Additional file 1: Figure S3).

With respect to independent predictors of mortality in the 413 patients with persistent or recurrent hypoperfusion, the utilization of dynamic tests of fluid responsiveness was independently associated to a decrease in mortality, adjusted by SOFA and Charlson scores, serum lactate and requirement of mechanical ventilation (Table 4). Calibration and discrimination of the model were adequate. The association between dynamic tests of fluid responsiveness and reduced mortality persisted in a mixed model with hospital as a

**Table 4:** Independent determinants of mortality according to logistic regression analysis

Variable	Odds ratio	[CI 95%]	*P*
Charlson score	1.21	[1.07–1.36]	0.002
SOFA score	1.16	[1.07–1.26]	< 0.0001
Serum lactate	1.21	[1.08–1.37]	0.001
Mechanical ventilation	12.2	[5.73–26.00]	< 0.0001
Dynamic tests of fluid responsiveness	0.37	[0.21–0.67]	0.001

Area under the ROC curve = 0.84. Hosmer–Lemeshow test = 0.45

random term (Additional file 1: Table S2).

## Discussion

This is the first study in Latin America characterizing patterns of fluid, vasopressor and inotrope use in the early resuscitation of patients with sepsis and septic shock. Our main finding was that the utilization of dynamic predictors of fluid responsiveness, after the initial fluid load, was independently associated with an improved outcome.

The SSC recommends administration of 30 mL/kg of fluids for patients with hypotension or with sepsis-induced hypoperfusion, within 3 h of identification—recently modified to a 1-h single bundle—to start resuscitation and management immediately [1, 16]. We observed good compliance with the 3 h-recommendation about administration of 30 mL/kg. In large controlled trials, fluid resuscitation followed the 3-h recommendation [17–19], but a recent observational study showed that only 47% of patients with septic shock received the initial fluid load [20]. These figures reflect the inconsistencies in implementation of bundles worldwide, regardless of the association with improved outcomes [2]. Yet, one retrospective study suggested that the beneficial effects are related to early antibiotics and not to volume expansion [21]. Furthermore, two clinical trials showed that aggressive fluid resuscitation is linked to increased mortality in both children and adults [22, 23]. Conversely, a recent study found that the failure to administer a bolus of 30 mL/kg of crystalloids within 3 h of sepsis onset was associated with increased in-hospital mortality, delayed hypotension, and increased ICU length of stay [24]. Finally, pilot-controlled studies failed to demonstrate benefits from restrictive fluid management within the first 24 h of resuscitation [25–28]. Therefore, early administration of 30 mL/kg of crystalloids in septic patients with hypoperfusion remains a controversial issue [29].

In our study, crystalloid solutions, especially NS and, to a lesser extent Ringer lactate, were the fluids most frequently indicated for intravascular volume expansion. Colloid solutions were occasionally used. In one French multicenter study, NS was the fluid of choice in 80% of fluid challenges, other crystalloids in 11%, and colloids were seldom indicated [30]. Another multicenter international study found that 74% of 2213 fluid challenges were administered using crystalloids (NS in 34% and balanced solutions in 40%), while 26% were administered with colloids (starches 11%, gelatins 9%, and albumin 5%) [12]. In an international survey of 3138 intensivists, NS was the most frequently considered acceptable (73%), followed by Ringer lactate and acetate (68 and 63%), albumin at 4% and 20% (43% and 36%, respectively), gelatins (32%) and starches (21%) [31].

Our results differ from those described in the multicenter international study [12]. The low utilization of colloid solutions (< 1%) is a direct result of the high cost in our country; however, this also reflects global trends related to nephrotoxicity of starches [32, 33] and failure of clinical trials demonstrating benefits from albumin use [34, 35]. Similar to our findings, NS was still the most commonly used solution in that study, despite recent evidence favoring balanced crystalloid solutions [36].

In accordance with SSC statements [1], norepinephrine was infused in 65% of patients in order to reach a MAP of at least 65 mm Hg. In 22% of them, norepinephrine was started before finishing the initial fluid bolus. These results are in line with those reported in a survey about the current use of vasopressors: 823 physicians from 82 countries responded that the persistence of arterial hypotension after the initial fluid load was the most frequent trigger for the administration of vasopressors (83%), and that norepinephrine was the drug of choice (97%) [37]. An expert panel recommended the early administration of norepinephrine to reach a MAP ≥ 65 mm Hg and suggested avoiding delays associated with the completion of the fluid load [37]. Notwithstanding, the optimal starting point for vasopressor administration remains controversial. The persistence of arterial hypotension has detrimental effects on outcomes and some studies suggest the advantages of early administration of vasopressors [38, 39], while others found an association between early administration and mortality [40, 41].

After the initial fluid load of 30 mL/kg and the titration of norepinephrine to reach a MAP of ≥ 65 mmHg, physicians recorded that more than half of patients still presented evidence of hypoperfusion. The diagnosis of hypoperfusion took into account clinical, hemodynamic, and biochemical variables. The most common criteria were tachycardia, oliguria, and hyperlactatemia. Although alterations in skin perfusion are a classic manifestation of shock, capillary-refill time and mottling were only assessed in about a quarter of patients. Capillary-refill time not only has relevant prognostic implications in septic shock, but is also a valuable resuscitation goal [42–44].

Even though insufficient fluid resuscitation can decrease tissue perfusion and affect mortality, a growing body of evidence suggests that, in septic shock, excessive fluid administration might be harmful [4–7]. Furthermore, only half of patients respond to fluid challenges with increases in cardiac output [45]. Central venous and pulmonary wedge pressures are misleading indicators of this response [46]. Instead, dynamic tests of fluid responsiveness, such as the respiratory variation of pulse pressure or the inferior vena cava distensibility, are suitable predictors [8]. Thus, the dynamic assessment of fluid responsiveness might optimize intravascular volume and contribute to the improvement of outcomes in septic shock [8]. In our study, all patients with persistent or recurrent hypoperfusion received additional fluid boluses after the initial fluid load. The decision was mainly based on the presence of tachycardia, hyperlactatemia, and oliguria. The dynamic assessment of fluid responsiveness, however, was only considered in 30% of patients. This figure is low but higher than the 22% recently reported in an international study [12]. An important finding of our study was that the use of dynamic tests of fluid responsiveness was independently associated with decreased mortality, even after adjusting for multiple confounders. For this analysis, we considered all dynamic tests as a whole. Although the tests are quite different from one another, they share the same underlying principle: to mimic the effect of a fluid bolus on stroke volume [8]. For this purpose, these tests take into account heart–lung interactions during mechanical ventilation or the response to postural changes.

Despite this rationale, small randomized controlled trials have failed to demonstrate that using dynamic tests to guide fluid resuscitation decreases mortality. In one study, 60 patients with septic shock who had received at least 25 mL/kg of crystalloids were allocated to intravascular volume expansion guided by either dynamic preload indices (pulse pressure variation and passive leg raising) or by central venous pressure [47]. The use of dynamic indices resulted in less fluid intake, but differences in mortality did not reach statistical significance (23 vs. 47%). In another study, fluid resuscitation based on the dynamic assessment of fluid responsiveness was compared to standard care in 82 patients who had received volume expansion with ≥ 30 mL/kg of crystalloids. Yet fluid balance or outcomes did not differ between groups [48]. Another study carried out in 122 septic patients on arrival at the emergency department showed that fluid resuscitation guided by the passive leg-raising maneuver did not improve outcomes [49]. In 101 patients with septic shock, the use of stroke volume variation decreased acute kidney injury, but did not improve other outcomes [50]. Passive leg-raising combined with transthoracic echocardiography improved lactate levels, pulmonary oxygenation and edema, and decreased hospital stay but had no effect on mortality [51]. Accordingly, a recent meta-analysis including four studies (365 patients) found no significant difference in mortality between septic patients resuscitated with a volume responsiveness-guided approach compared with standard care [52]. Nevertheless, adequately powered clinical trials are lacking.

Our study has limitations. The observational design does not allow drawing definitive conclusions. In addition, a selection bias related to voluntary participation is possible. As this was not a clinical trial, the time-points of assessment of hypoperfusion were not pre-specified, but were left to physician evaluation. Moreover, the amount of fluid boluses are not available. It is possible that the use of dynamic tests of fluid responsiveness might act as a confounder of physicians’ expertise and/or better-equipped ICUs, hence the decreased mortality could be a consequence of these two factors. However, an independent effect of the different centers on mortality was not found in the mixed-effects regression analysis.

## Conclusions

In this prospective observational study assessing the characteristics of resuscitation in septic patients in Argentina, a LMIC country, the prevalent use of an initial fluid load with 0.9% normal saline solution and the early administration of norepinephrine reflect current worldwide practices. More than half of patients developed persistent or recurrent hypoperfusion after initial resuscitation with 30 mL/kg of fluids, which required further volume expansion. In this setting, the assessment of fluid responsiveness with dynamic tests to guide fluid resuscitation was independently associated with decreased mortality. Large controlled trials are required to confirm this observation.

## Supplementary information

**Additional file 1. Table S1.** Epidemiological and hemodynamic data in the entire cohort, and in survivors and nonsurvivors. **Table S2.** Independent determinants of mortality according to a mixed-effect model, in which hospitals (centers) were added as the random term. **Figure S1.** Frequency of the variables utilized as a guide for the additional administration of fluids after the initial bolus. **Figure S2.** Vasopressors and/or inotropes used in the resuscitation of septic patients. **Figure S3.** Type of solution used for the initial fluid bolus of 30 mL/kg. **Tests used for the dynamic assessment of fluid responsiveness**.

## Data Availability

The datasets used and/or analyzed during the current study are available from the corresponding author on reasonable request.

## Abbreviations

SSC: Surviving Sepsis Campaign
MAP: Mean arterial pressure
SATI: Sociedad Argentina de Terapia Intensiva
LMIC: Low and middle-income countries
NS: Normal saline
ROC: Receiver operating characteristic
