# Supplementary material for: Characteristics of resuscitation, and association between use of dynamic tests of fluid responsiveness and outcomes in septic patients: results of a multicenter prospective cohort study in Argentina
Source: Ann Intensive Care. 2020 Apr 15;10:40. doi: 10.1186/s13613-020-00659-7 (PMC7158970; doi:10.1186/s13613-020-00659-7)
Supplement: Supplementary file 1 — Additional file 1. Table S1. Epidemiological and hemodynamic data in the entire cohort, and in survivors and nonsurvivors. Table S2. Independent determinants of mortality according to a mixed-effect model, in which hospitals (centers) were added as the random term. Figure S1. Frequency of the variables utilized as a guide for the additional administration of fluids after the initial bolus. Figure S2. Vasopressors and/or inotropes used in the resuscitation of septic patients. Figure S3. Type of solution used for the initial fluid bolus of 30 mL/kg. Tests used for the dynamic assessment of fluid responsiveness. [file 13613_2020_659_MOESM1_ESM.docx]

**Additional file 1**

**CHARACTERISTICS OF RESUSCITATION, AND ASSOCIATION BETWEEN USE OF DYNAMIC TESTS OF FLUID RESPONSIVENESS AND OUTCOMES IN SEPTIC PATIENTS: RESULTS OF A MULTICENTER PROSPECTIVE COHORT STUDY IN ARGENTINA**

Arnaldo Dubin^1*^, Cecilia Loudet^2^, Vanina Kanoore Edul^3^, Javier Osatnik^4^, Fernando Ríos^5^, Daniela Vásquez^6^, Mario Pozo^7^, Bernardo Lattanzio^8^, Fernando Pálizas^7^, Francisco Klein^9^, Damián Piezny^5^; Paolo N. Rubatto Birri^1^, Graciela Tuhay^9^, Analía García^10^, Analía Santamaría^11^, Graciela Zakalik^12^, Cecilia González^13^, and Elisa Estenssoro^2^; on behalf of the investigators of the SATISEPSIS group.

^1^Sanatorio Otamendi, Ciudad Autónoma de Buenos Aires, Argentina. ^2^Hospital Interzonal de Agudos San Martin de La Plata, La Plata, Buenos Aires, Argentina. ^3^Hospital Juan A Fernández, Ciudad Autónoma de Buenos Aires, Argentina. ^4^Hospital Alemán, Ciudad Autónoma de Buenos Aires, Argentina. ^5^Hospital Alejandro Posadas, El Palomar, Buenos Aires, Argentina. ^6^Sanatorio Anchorena, Ciudad Autónoma de Buenos Aires, Argentina. ^7^Clínica Bazterrica, Ciudad Autónoma de Buenos Aires, Argentina. ^8^Clínica Santa Isabel, Ciudad Autónoma de Buenos Aires, Argentina. ^9^Hospital Universitario Fundación Favaloro, Ciudad Autónoma de Buenos Aires, Argentina. ^10^Hospital Misericordia, Ciudad de Córdoba, Córdoba, Argentina. ^11^Sanatorio de la Trinidad Mitre, Ciudad Autónoma de Buenos Aires, Argentina. ^12^Hospital Lagomaggiore, Ciudad de Mendoza, Mendoza, Argentina. ^13^Sanatorio Parque, Rosario, Santa Fe, Argentina.

^*^Corresponding author:

Arnaldo Dubin

arnaldodubin@gmail.com

Servicio de Terapia Intensiva, Sanatorio Otamendi, Azcuénaga 870, C1115 AAB, Ciudad Autónoma de Buenos Aires, Argentina

| **Table S1. Epidemiological and hemodynamic data in the entire cohort, and in survivors and nonsurvivors.** | | | | |
| --- | --- | --- | --- | --- |
|  | **All**  **(n=787)** | **Survivors**  **(n=493)** | **Nonsurvivors**  **(n=294)** | ***P*** |
| **Age (years)** | 62 [46-74] | 60 [43-74] | 64 [54-74] | <0.001 |
| **Gender (female)** | 346/787 (44) | 227/493 (46) | 119/294 (40) | 0.09 |
| **Charlson score** | 2 [0-3] | 1 [0-3] | 2 [1-4] | <0.0001 |
| **APACHE II score** | 19±8 | 17±7 | 23±9 | <0.0001 |
| **SOFA 24-h score** | 7 [3-18] | 10 [4-20] | 6 [2-16] | <0.0002 |
| **Lactate (mmol/L)** | 2.1 [1.3-3.4] | 1.9 [1.1-3.0] | 2.4 [1.6-4.1] | <0.0001 |
| **Mean arterial pressure <65 mm Hg and/or hypoperfusion** | 573/787 (73) | 331/492 (67) | 242/295 (82) | <0.0001 |
| **Initial fluid load of 30 mL/kg** | 573/787 (73) | 341/491 (69) | 232/295 (79) | <0.005 |
| **Type of solution** |  |  |  |  |
| **NaCl 0.9%** | 418/784 (53.32) | 234/490 (47.76) | 184/294 (62.59) | <0.0001 |
| **Ringer lactate** | 278/782 (35.55) | 168/488 (34.43) | 110/294 (37.41) | 0.398 |
| **Mean arterial pressure >65 mm Hg after initial fluid load** | 401/787 (51) | 275/493 (56) | 126/294 (43) | <0.0001 |
| **Use of norepinephrine** | 514/787 (65) | 270/493 (55) | 244/294 (83) | <0.0001 |
| **Norepinephrine dose (μg/kg/min)** | 0.30 [0.15-0.60] | 0.25 [0.16-0.50] | 0.60 [0.28-1.00] | <0.0001 |
| **Use of dobutamine** | 44/787 (6) | 16/493 (3) | 28/294 (10) | <0.0002 |
| **Dose of dobutamine (μg/kg/min)** | 5.0 [4.0-9.0] | 5.0 [5.0-8.7] | 5.0 [5.0-9.5] | 0.92 |
| **Assessment of the response to initial fluid load** |  |  |  |  |
| **Mean arterial pressure** | 527/783 (67) | 314/490 (64) | 213/293 (73) | 0.013 |
| **Heart rate** | 392/782 (50) | 234/489 (48) | 158/293 (54) | 0.10 |
| **Central venous pressure** | 179/780 (23) | 100/488 (20) | 79/292 (274) | 0.035 |
| **Lactate** | 424/781 (54) | 243/488 (50) | 181/293 (62) | <0.001 |
| **Central venous O_2_ saturation** | 281/781 (36) | 165/489 (34) | 116/292 (40) | 0.092 |
| **Central venous-arterial PCO_2_ difference** | 226/781 (29) | 151/489 (27) | 95/292 (32) | 0.087 |
| **Urine output** | 434/782 (55) | 271/489 (55) | 163/293 (56) | 0.954 |
| **Capillary refill time** | 204/780 (26) | 122/487 (25) | 82/293 (28) | 0.366 |
| **Mottling** | 188/781 (24) | 107/488 (22) | 81/293 (28) | 0.070 |
| **Persistent or recurrent hypoperfusion after initial fluid load*** | 413/783 (53) | 216/490 (44) | 197/293 (67) | <0.0001 |
| **Length of mechanical ventilation (days)** | 4 [0-14] | 2 [0-8] | 5 [2-11] | <0.0001 |
| **ICU length of stay (days)** | 8 [4-10] | 6 [3-8] | 9 [7-11] | <0.0001 |
| **Hospital length of stay (days)** | 17 [8-33] | 23 [13-40] | 10 [3-23] | <0.0001 |
| **Septic shock on admission** | 323/787 (41) | 182/493(37) | 141/294 (48) | <0.0001 |
| **On admission category:(medical/programmed surgery/emergency surgery)** | 583/52/151  (74/7/19) | 228/18/47  (78/6/16) | 355/34/104  (72/7/21) | 0.18 |
| Data are shown as number (percentage), median [IQR] or mean ± SD.  *22% of these patients also received norepinephrine. | | | | |

| **Table S2. Independent determinants of mortality according to a mixed-effect model, in which hospitals (centers) were added as the random term.** | | | | | |
| --- | --- | --- | --- | --- | --- |
| **Variable** | **Coefficient** | **SE** | **z** | **P>\|z\|** | **[CI 95%]** |
| **SOFA score** | 0.025 | 0.006 | 4.04 | 0.0001 | [0.013-0.038] |
| **Serum lactate** | 0.029 | 0.009 | 3.20 | 0.001 | [0.011-0.0478] |
| **Charlson score** | 0.033 | 0.009 | 3.55 | 0.000 | [0.015-0.0512] |
| **Mechanical ventilation** | 0.422 | 0.052 | 8.17 | 0.000 | [0.321-0.523] |
| **Dynamic tests of fluid responsiveness** | -0.163 | 0.053 | -3.04 | 0.002 | [-0.268--0.058] |
| **constant** | -0.176 | 0.063 | -2.79 | 0.005 | [-0.300--0.052] |

**Random-effects Parameters Estimate SE [95% CI]**

**Hospital: Independent**

Sd (testdyn) 0.076 0.094 [0.007-0.8634]

Sd (_cons) 0.058 0.049 [0.011-0.306]

---------------------------------------------------------------------------------------------------------------------

Sd (Residual) 0.4003329 0.0161333 [0.3699288-0.4332359]

---------------------------------------------------------------------------------------------------------------------

LR test vs. linear regression: chi^2^(2) = 1.04, Prob > chi^2^ = 0.5936

Figure S1. Frequency of the variables utilized as a guide for the additional administration of fluids after the initial bolus.

Abbreviations: S_cv_O_2_, central venous O_2_ saturation; P_cv-a_CO_2_, central venous-arterial PCO_2_ difference; MAP, mean arterial pressure; CVP, central venous pressure; CRT, capillary-refill time. Dynamic assessment of fluid responsiveness include the following tests: the respiratory variation of arterial pulse pressure, systolic volume, and pulse oximetry plethysmographic waveforms; the respiratory variation of inferior vena cava diameter; and the passive leg raising maneuver.

Figure S2. Vasopressors and/or inotropes used in the resuscitation of septic patients.

Figure S3. Type of solution used for the initial fluid bolus of 30 mL/kg.

**TESTS USED FOR THE DYNAMIC ASSESSMENT OF FLUID RESPONSIVENESS**

The participating centers used different tests for the dynamic assessment of fluid responsiveness. There was a wide heterogeneity in their availability and utilization. Although the use of the tests was not protocolized, some definitions and cutoffs values were suggested.

**Respiratory variation of arterial pulse pressure (PPV):** Measurable in patients with arterial lines connected to multiparameter monitors with proper software. Patients should be mechanically ventilated patients without spontaneous efforts, regular cardiac rate, and static respiratory compliance >30 mL/cmH_2_O. Patients with PPV ≥13% should be considered fluid responsive. If the patient is under protective mechanical ventilation, tidal volume should be increased transiently to 8 ml/kg of body weight.

**Respiratory variation of stroke volume:** Measurable in patients with arterial lines connected to devices that measure stroke volume by analysis of pulse pulse contour, either calibrated or noncalibrated. The same limitations of PPV should be considered. The cutoff value is 12%.

**Respiratory variation of pulse oximetry plethysmographic waveform amplitude:** Available in some pulse oximeter systems. The same limitations of PPV should be considered. The cutoff value is 16%.

**Respiratory variation of inferior vena cava diameter:** Maximum and minimum inferior vena cava diameters should be measured in M-mode, using the subxiphoid view. The distensibility index is calculated in mechanically ventilated patients as (maximum diameter-minimum diameter)/minimum diameter. The cutoff value is 18%.

**Passive leg raising maneuver:** Suitable in spontaneously or mechanically ventilated patients. Starting from the semi-recumbent position with the trunk at 45°, legs are elevated by adjusting the bed. Touching the patient should be avoided. Continuous measurements of cardiac output (arterial pulse contour analysis, echocardiography, esophageal Doppler) or end-tidal PCO_2_ should be performed to show increases ≥10% or ≥5%, respectively. Reassessment in semi-recumbent position should be done to confirm the return to baseline.

**End-expiratory occlusion test:** Measurable in mechanically ventilated patients with real-time measurements of cardiac output. An end-expiratory pause ≥15 s should be performed without patient’s efforts. Cutoff value is 5%.

**REFERENCES**

1. Michard F, Boussat S, Chemla D, Anguel N, Mercat A, Lecarpentier Y, et al. Relation between respiratory changes in arterial pulse pressure and fluid responsiveness in septic patients with acute circulatory failure. Am J Respir Crit Care Med. 2000;162:134-8.
2. Myatra SN, Prabu NR, Divatia JV, Monnet X, Kulkarni AP, Teboul JL. The Changes in Pulse Pressure Variation or Stroke Volume Variation After a "Tidal Volume Challenge" Reliably Predict Fluid Responsiveness During Low Tidal Volume Ventilation. Crit Care Med. 2017;45:415-21.
3. Biais M, Nouette-Gaulain K, Roullet S, Quinart A, Revel P, Sztark F. A comparison of stroke volume variation measured by Vigileo/FloTrac system and aortic Doppler echocardiography. Anesth Analg. 2009;109:466-9.
4. Monnet X, Teboul JL. Assessment of fluid responsiveness: recent advances. Curr Opin Crit Care. 2018;24:190-5.
5. Loupec T, Nanadoumgar H, Frasca D, Petitpas F, Laksiri L, Baudouin D, et al. Pleth variability index predicts fluid responsiveness in critically ill patients. Crit Care Med. 2011;39:294-9.
6. Barbier C, Loubières Y, Schmit C, Hayon J, Ricôme JL, Jardin F, et al. Respiratory changes in inferior vena cava diameter are helpful in predicting fluid responsiveness in ventilated septic patients. Intensive Care Med. 2004;30:1740-6.
7. Feissel M, Michard F, Faller JP, Teboul JL. The respiratory variation in inferior vena cava diameter as a guide to fluid therapy. Intensive Care Med 2004;30:1834-7.
8. Monnet X, Rienzo M, Osman D, Anguel N, Richard C, Pinsky MR, et al. Passive leg raising predicts fluid responsiveness in the critically ill. Crit Care Med. 2006;34:1402-7.
9. Lamia B, Ochagavia A, Monnet X, Chemla D, Richard C, Teboul JL. Echocardiographic prediction of volume responsiveness in critically ill patients with spontaneously breathing activity. Intensive Care Med. 2007;33:1125-32.
10. Monge García MI, Gil Cano A, Gracia Romero M, Monterroso Pintado R, Pérez Madueño V, Díaz Monrové JC. Non-invasive assessment of fluid responsiveness by changes in partial end-tidal CO_2_ pressure during a passive leg-raising maneuver. Ann Intensive Care. 2012;2:9.
